# Integrating habit science and learning theory to promote maintenance of behavior change: does adding text messages to a habit-based sleep health intervention (HABITs) improve outcomes for eveningness chronotype young adults? Study protocol for a randomized controlled trial

**DOI:** 10.1186/s13063-024-08599-4

**Published:** 2024-11-20

**Authors:** Marlen Diaz, Estephania Ovalle Patino, Sophia Oliver, Sondra S. Tiab, Nena Salazar, Jiyoung Song, Lu Dong, Laurel D. Sarfan, Eli S. Susman, Emma R. Agnew, Benjamin Gardner, Allison G. Harvey

**Affiliations:** 1 Department of Psychology, University of California, Berkeley, CA USA; 2 Behavioral and Policy Sciences, RAND Corporation, Santa Monica, CA USA; 3 Department of Research and Evaluation, Kaiser Permanente Southern California, Pasadena, CA USA; 4 School of Psychology, University of Surrey, Guildford, Surrey UK

**Keywords:** Transdiagnostic, Sleep, Circadian, Habits, Health, Text message, Intervention

## Abstract

**Background:**

Eveningness chronotype—the tendency for later sleep and wake times—arises from a confluence of psychosocial, behavioral, and biological factors. With the onset and progression of puberty, many young people develop an eveningness chronotype, which remains prevalent through the transition into adulthood. Eveningness has been associated with increased risk for poorer health. While eveningness is modifiable, maintaining the necessary behavior changes can be challenging. The science on habits demonstrates that habit formation is a key mechanism for maintaining behavior change over time. Learning theory offers schedules of reinforcement that also hold promise for enhancing the maintenance of behavior change. The present study will evaluate the Habit-based Sleep Health Intervention (HABITs)—which combines the Transdiagnostic Intervention for Sleep and Circadian Dysfunction (TranS-C) with the science of habits—and a text message intervention informed by learning theory to attempt to sustainably modify the contributors to eveningness among young adults (18–30 years of age).

**Methods:**

Participants (*N* = 160) will be randomly allocated to HABITs and HABITs + Texts. Both interventions include HABITs which involves three 50-min sessions followed by six 30-min sessions. Alongside the latter six sessions, HABITs + Texts will concurrently receive the text message intervention. Aims 1–3 will compare HABITs + Texts to HABITs on improvements in the outcomes of (1) utilization of sleep health behaviors and habit formation, (2) sleep and circadian functioning, and (3) functioning in five health-relevant domains, in the short (post-treatment) and longer (6-month and 12-month follow-up) term. Exploratory analysis will (1) compare HABITs and HABITs + Texts on (a) if sleep health behavior habit formation mediates the effects of intervention on improvement in outcomes and (b) if intervention effects are moderated by select variables, and (2) to evaluate if HABITs (regardless of the text message intervention) is associated with an improvement in outcomes in the short and longer term.

**Discussion:**

This study has the potential to advance knowledge on (1) the value of leveraging the science of habits and learning theory in behavior change interventions, (2) the use of a low-cost and efficient intervention for habit formation and maintenance, (3) interventions that address eveningness chronotype, and (4) processes related to behavior change during emerging adulthood.

**Trial registration:**

Clinicaltrials.gov NCT05167695. Registered on December 22, 2021.

**Supplementary Information:**

The online version contains supplementary material available at 10.1186/s13063-024-08599-4.

## Background

People who exhibit an eveningness chronotype prefer a delayed sleep–wake schedule, typically going to sleep later and waking up later [1–3]. Eveningness has been associated with increased risk for adverse health consequences across five health-relevant domains including the emotional [4–6], cognitive [7–9], behavioral [10–13], social [14], and physical [15, 16] domains. Although much of this research has been cross-sectional, several longitudinal studies have reported the same pattern of findings [17–20]. Despite the occasional non-replication [21, 22], the risk for adverse health consequences of eveningness are concerning.

Eveningness arises from a confluence of psychosocial, behavioral, and biological factors. For instance, many youth develop eveningness with the onset and progression of puberty [23–25]. Eveningness typically reaches a peak around 16–20 years of age [25–27]. This age group marks the beginning of an important developmental period—emerging adulthood—typically covering 18–30 years of age [28]. The developmental milestones during this phase of life shift priorities toward self-sufficiency and personal responsibility, which are supported by the formation of helpful behaviors and dismantling of unhelpful behaviors [29, 30]. The goal of the present study is to evaluate an approach to facilitate behavior change that modifies the psychosocial and behavioral contributors maintaining eveningness in young adults [31].

There are promising signals that the psychosocial and behavioral contributors maintaining eveningness may be modifiable [31–34]. For example, the Transdiagnostic Intervention for Sleep and Circadian Dysfunction (TranS-C) [35], aims to modify the psychosocial and behavioral contributors to eveningness and has exhibited promising results on selected sleep, circadian, and health outcomes [34, 36–38], relative to Psychoeducation. However, Gumport et al. [39] assessed the frequency with which youth who participated in this study were utilizing the sleep health behaviors they learned in TranS-C at the 6-month and 12-month follow-up and the result was disappointing. Participants reported using the sleep health behaviors only “occasionally” at 6-month and 12-month follow-up. In a long-term follow-up, an average of 8 years after the intervention, TranS-C participants who were utilizing sleep health behaviors and who had formed sleep health habits had better outcomes (i.e., lower levels of eveningness) [40]. These findings raise a new empirical question; namely, would outcomes following the receipt of interventions, like TranS-C, improve if knowledge relating to behavior change—such as the science of habits and learning theory—could be leveraged to improve the utilization of treatment elements?

The accumulated body of science on habits offers exciting opportunities for improving interventions like TranS-C [41–43]. “Habit” is defined as a process whereby contextual cues, via repetition and reward, come to automatically trigger an impulse to engage in a specific behavior [44–46]. “Habitual behavior” is defined as any behavior triggered by the habit process. To form a habit, a behavior must be repeated after exposure to a stable contextual cue [47, 48]. This strengthens a behavior-context association, to the extent that subsequently encountering the contextual cue triggers an unconscious impulse to engage in the habitual behavior, which in turn translates into the activation of behavior, with little prior thought or conscious intention [41]. The impact of repetition on habit formation is strengthened by rewards, such that repeating more rewarding behaviors causes more rapid or stronger habit formation compared to less rewarding behaviors [49, 50]. Overall, habit promotes frequent engagement in behavior [51–54]. By virtue of their automaticity, habitual behaviors are thought to persist over time, potentially even when people lack motivation to perform them [55]. Habit formation is thus a key mechanism for maintaining behavior change [56]. By integrating the science on habits with behavior change interventions like TranS-C, the aim of the study described herein is to help participants turn new behaviors into habits and thereby increase the benefits of an intervention in both the short and longer term [42, 55]

Learning theory offers predictions about schedules of reinforcement that may further enhance the maintenance of behavior change [57]. Learning theory can be used to inform the timing and frequency (i.e., schedule) of cues and rewards to ensure that a habit formation process is maximally potent. We hypothesize that text messages can scaffold habit formation, particularly if informed by learning theory and the science of habits. Specifically, we will use text messages to promote the development of automaticity via the strengthening of contextual cues, formalizing self-monitoring, and by adopting a schedule of reinforcement that maximizes the chance for sustained behavior change. The schedule of reinforcement selected will start with continuous reinforcement and be followed by a switch to partial reinforcement and an “expanding-spaced” schedule. The rationale for this choice is grounded in the evidence that this sequence of reinforcement schedules promote resistance to extinction [58, 59]. Text messages were selected because mobile phones are personal and constantly accessible, with text messages being popular, particularly among young adults [60]. Texts catch an individual’s attention, are likely to be read within minutes of being received, and tend to be carefully considered [61]. Also, text messages are increasingly yielding positive results when used to deliver or complement health interventions [62, 63] and to promote habit formation [64]. There is growing evidence that text messaging interventions may improve sleep-related outcomes, but findings have been mixed [36, 65]. Finally, a text message intervention has a low-cost and is efficient. Thus, text messages may help with habit formation and bolstering the maintenance of behavior change [62–64].

In the present study, we will test a sleep-health intervention that leverages the science of habits: the Habit-based Sleep Health Intervention (HABITs). HABITs aims to modify the psychosocial and behavioral contributors to eveningness by supporting the utilization of sleep-health behaviors via habit formation. HABITs combines TranS-C [35] and the science of habits [41, 55, 66, 67]. Drawing from the latter, HABITs incorporates establishing a contextual cue [29], encouraging repetition [49], building in reinforcers [68, 69], allowing time—as habits can be slow to develop and change [29]—and accurate measurement and assessment of the formation of habitual behavior [45, 70, 71]. Importantly, this study is grounded in the idea that leveraging the science of habits could markedly improve outcomes from a broad range of evidence-based psychological treatments, like TranS-C [42].

In addition to testing HABITs, we will evaluate if adding a novel text message intervention improves the utilization of sleep health behaviors, habit formation, and outcomes. In the present study, the text message intervention was theoretically derived using the science of habits [41], learning theory [57], and the Behavior Change Wheel [67]. The Behavior Change Wheel provides a systematic approach to enhance capability, opportunity, and motivation to improve sleep health behaviors (COM-B). Two team members trained in Behavior Change Wheel methods used the COM-B model, determined the intervention functions, and selected Behavior Change Techniques [72] to guide the derivation of the text messages. The text message intervention was further developed through focus groups, a pilot study, and recommendations for developing, pre-testing, tailoring, and personalizing the text message interventions [61, 73]. The text message intervention consists of three types of texts to support habit formation (examples can be found in the Methods section). First, the cue texts will be included to facilitate the process of establishing and encoding the chosen contextual cue while pairing it with the selected behavior. This reminder of the contextual cue should come to trigger the behavior, as proposed by Pavlovian conditioning [48, 74]. Second, the self-monitoring texts will be used to determine when a reward text is warranted, this is also a form of progress monitoring and thus an intervention in and of itself [75, 76]. Third, the reward texts can have a profound impact on the frequency and the longevity of a behavior [50, 57, 69].

## Aims

This randomized controlled two-arm parallel group superiority study with 1:1 allocation ratio will be conducted with young adults who are 18–30 years of age, exhibit an eveningness chronotype, and demonstrate elevated symptoms in one of the five health domains. Participants will be randomized (1:1) to HABITs alone or HABITs plus Text Message Intervention (HABITs + Texts). There are three aims.

Aim 1 is to assess if adding a text message intervention to HABITs improves sleep health behaviors in the short (post-treatment) and longer term (6-month and 12-month follow-up), relative to HABITs alone. Hypothesis 1 is that, relative to HABITs, participants in HABITs + Texts will (a) report utilizing more sleep health behaviors and (b) establish stronger sleep health behavior habits. We hypothesize that these effects (1a and 1b) will be observed from pre-treatment to post-treatment, pre-treatment to 6-month follow-up, and pre-treatment to 12-month follow-up.

Aim 2 is to assess if adding a text message intervention to HABITs improves sleep and circadian functioning in the short (post-treatment) and longer term (6-month and 12-month follow-up), relative to HABITs alone. Hypothesis 2 is that, relative to HABITs, participants in HABITs + Texts will exhibit improved sleep and circadian functioning. We hypothesize that this effect will be observed from pre-treatment to post-treatment, pre-treatment to 6-month follow-up, and pre-treatment to 12-month follow-up.

Aim 3 is to assess if adding a text message intervention to HABITs improves functioning in the five health-relevant domains in the short (post-treatment) and longer term (6-month and 12-month follow-up), relative to HABITs alone. Hypothesis 3 is that, relative to HABITs, participants in HABITs + Texts will exhibit lower health risk. We hypothesize that this effect will be observed from pre-treatment to post-treatment, pre-treatment to 6-month follow-up, and pre-treatment to 12-month follow-up.

As an Exploratory Aim 1, comparing HABITs and HABITs + Texts, we will examine (a) if the strength of sleep health behavior habit formation mediates the effects of the intervention (specifically by adding the text intervention to HABITs) on improvement in sleep and circadian outcomes, and health-relevant domain outcomes, and (b) if intervention effects are moderated by selected variables (i.e., age, sex, and socioeconomic status).

Lastly, should the HABITs versus HABITs + Texts intervention effects be non-significant, we will conduct Exploratory Aim 2: an analyses combining the two randomized groups (HABITs and HABITs + Texts) to evaluate if HABITs (regardless of whether the text message intervention is added) is associated with an improvement in the utilization of sleep health behaviors, sleep health behavior habits, sleep and circadian outcomes, and health-relevant domain outcomes in the short (pre-treatment to post-treatment) and longer term (pre-treatment to 6-month and 12-month follow-up).

## Method

**Fig. 1:**
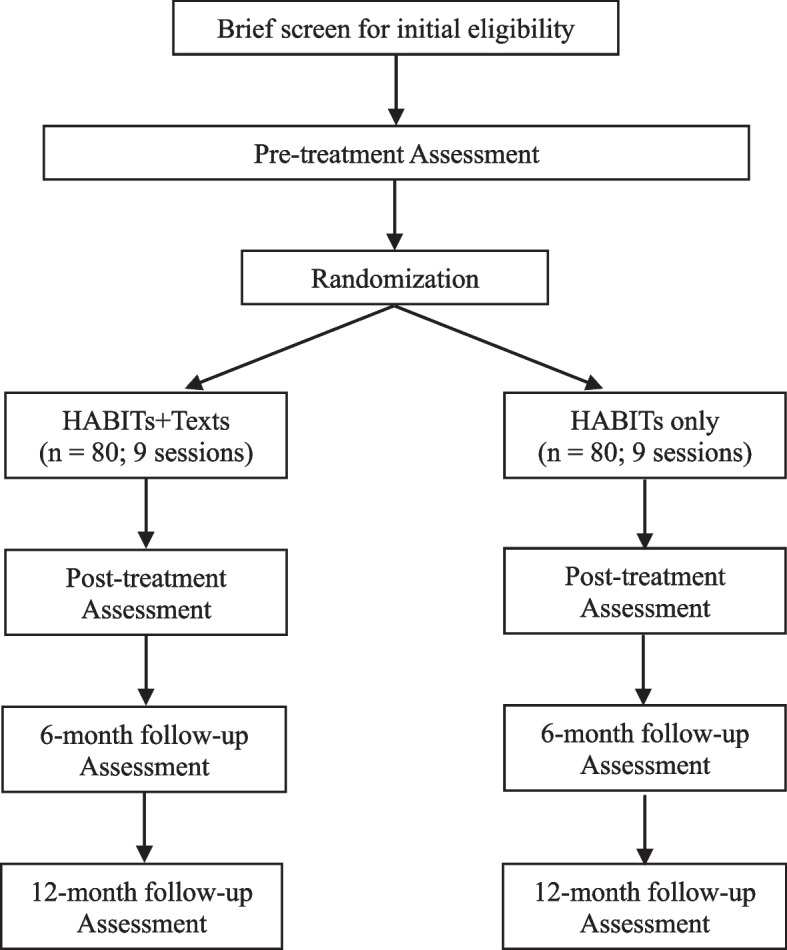
Anticipated study flow

This study was preregistered on clinicaltrials.gov (identifier: NCT05167695) and received approval from the Committee for the Protection of Human Subjects (CPHS) at the University of California, Berkeley (UCB) (2021–06-14409). Any protocol changes will be submitted to clinicaltrials.gov and CPHS. The research team will communicate relevant changes in appropriate publications (e.g., see Changes to Preregistration section below). If there are too many findings to reasonably interpret in one paper, we may separate some of the findings into two or more papers. This research is funded by the National Institute of Child Health and Human Development (NICHD; R01HD071065). The present protocol used the SPIRIT reporting guidelines [77] (see SPIRIT checklist in Additional file 1 and Fig. 1).

### Participants

The inclusion criteria will be as follows: (1) age between 18 and 30, (2) risk for eveningness chronotype: scoring less than or equal to 26 on the Composite Scale of Morningness or a mid-point of sleep later than 4:30 a.m. for 18–24 years of age and 3:50 a.m. for 25–30 years of age on work-free/weekend days over the past month or night-to-night variation in sleep and wake times across one month of 2 h or more, (3) at risk in one of the five health domains: the emotional, cognitive, behavioral, social, or physical domain (at risk is defined as scoring 4 or higher on one item from the Adapted Version of the Work and Social Adjustment Scale), (4) English language fluency, (5) able and willing to give informed consent, and (6) if taking medication for sleep, the dose and frequency of use must have been stable for at least 4 weeks.

Participants will be excluded if they meet any of the following criteria: (1) presence of substance abuse/dependence, mental illness, physical illness, suicidality, or developmental disorder only if it makes participation in the study unfeasible or if there is a significant risk of harm and/or decompensation if treatment of that comorbid condition is delayed due to participating in this study, (2) evidence of sleep apnea, restless legs, or periodic limb movements during sleep (participants presenting with provisional diagnoses of any of these disorders will be referred for a non-study polysomnography evaluation and will be enrolled only if the diagnosis is disconfirmed or if the disorder is treated), (3) night shift worker where the shift is scheduled between the hours of midnight to 6:00 a.m. more than two nights per week, and (4) pregnancy or breast-feeding.

### Intervention

Two variations of the Habit-based Sleep Health Intervention (HABITs) will be tested: HABITs alone and HABITs + Texts. For both variations, HABITs will be delivered across three 50-min weekly sessions followed by six 30-min weekly sessions. The dose of nine weekly sessions^1^ was derived based on prior research and the amount of material that needs to be covered to maximize successful habit formation. The difference between the two intervention arms is that HABITs + Texts will receive a text message intervention for 6 weeks^2^ following session 3 through session 9, while the HABITs intervention arm will not receive the text message intervention. See Table 1 for a description of the intervention arms.

**Table 1:** Description of intervention arms: HABITs and HABITs + Texts

	**HABITs**	**HABITs + Texts**
**Sessions 1–3**	**Three 50-min sessions, consisting of** ^**a**^ **:** - Sleep diary review - Sleep and circadian science. For example: o Regularizing bed and wake times o Devising a rise-up routine o Devising a wind-down routine o Reducing sleep related worry/anxiety (i.e., relax the mind strategies) o Improving sleep efficiency (i.e., stimulus control and sleep restriction) o Improving daytime functioning - Habit science. For example: o Identifying contextual cues, the importance of repetition, and identifying rewards o Dismantle unhelpful habits by identifying contextual cues, rewards, and methods (i.e., substitution, removing or curtailing the activity, or re-organizing the activity) - Case conceptualization with script elicitation to derive habit bundle(s) o Typically, 1–2 bundles (i.e., primary and secondary; for examples, see Table 2) - Motivational enhancement - Home projects	
**Sessions 4–9**	**Six 30-min sessions, consisting of** ^**a**^ **:** - Reviewing and refining sleep habit bundle(s) - Assessing progress and obstacles - Sleep and circadian science - Habit science - Reinforcing contextual cues and motivations - Maintenance and relapse education	
	**No text messaging**	**Six weeks of text messaging, consisting of:** - Cues - Self-monitoring - Rewards

^a^Same procedure across both treatment arms

#### HABITs

HABITs combines TranS-C [35] and the science of habits [41, 66, 67]. HABITs sessions 1–3 will focus on (1) education on sleep and circadian science, (2) education on the science of habits, (3) deriving habit bundle(s) goals, (4) collaboratively developing case conceptualization combined with script elicitation, and (5) motivational enhancement. These five elements, described in more detail below, are “rolling interventions,” which will be flexibly revisited in sessions 4–9, where the focus will be on forming helpful sleep habits and dismantling unhelpful sleep habits. Each session will begin with an agenda and end by devising home projects to complete between sessions.

##### Sleep and circadian science

Information will be incorporated session-by-session to provide a strong rationale for targeting collaboratively identified sleep health behaviors, which will become the “habit bundle(s).” Topics to be covered include the value of regularizing bed and wake times, devising a rise-up routine, devising a wind-down routine, reducing sleep-related worry/anxiety (i.e., relax the mind strategies), improving sleep efficiency (i.e., stimulus control and sleep restriction), and improving daytime functioning [35].

##### Science of habits

Information on the science of habits [41, 47, 67] will be incorporated session-by-session. Topics to be covered include identifying contextual cues, the importance of repetition, and identifying rewards—the keys to forming habits. In addition, information will be offered on how to dismantle unhelpful habits as these types of behaviors may interfere with the pursuit of new helpful behaviors [55].

##### Case conceptualization and script elicitation to derive habit bundle(s) goals

Using the assessment materials, sleep diary, the participant’s primary complaint(s), and the case conceptualization, the goal “habit bundle(s)” are collaboratively derived. We focus on habit *bundles* because sleep health habits typically consist of several behaviors. For examples, see Table 2. Typically, 1–2 habit bundle(s) are identified for each participant. We label the bundle addressing the participant’s primary complaint, as the “primary habit bundle.” The second is referred to as the “secondary habit bundle.” The bundle(s) include helpful and unhelpful habits in the areas of rise-up habits, wind-down habits (i.e., sleep-onset), wake after sleep onset habits, sleep efficiency habits, and daytime habits (for examples, see Table 2). Thus, for the habit bundle(s), participants focus on helpful habits they want to build and unhelpful habits they want to dismantle. Habit bundle(s) and specific behaviors vary from participant to participant.

**Table 2:** Examples of unhelpful and helpful habit bundles

Unhelpful habits	Helpful habits
**Rise-Up Habit Bundle**	
Snooze the morning alarm	Wake-up using an alarm clock
Stay in bed after waking up	Open curtains
Avoid turning on lights or opening curtains	Do stretches to get through the sleep inertia
Think about how much I am dreading the day	No snoozing
Wake-up much later on weekends	Get up within 10 min of the alarm clock
**Wind-down Habit Bundle**	
Worry about things that happened today	Dim the lights about an hour before bed
Engage in social media in bed	Turn off technology
Playing video games or watching a movie in bed	Engage in a wind-down activity
Bright lights on late into the night	Stimulus control if unable to sleep
Much later bedtimes on weekends	Similar bedtime from weekdays to weekends
**Wake-After Sleep-Onset Habit Bundle**	
Watch movie or check social media on my tablet	Stay in darkness or dim lights
Worry about the day or tomorrow	Relax the Mind strategies
Get up for a snack	Stimulus control if unable to get to sleep
**Sleep Efficiency Habit Bundle**	
Spend more time in bed than actually sleeping	Sleep restriction or Sleep compression
**Daytime Habit Bundle**	
Cancel appointments or plans	Generate energy
Drink caffeine and/or alcohol late into day	Socialize as planned
Nap	Work as planned

Combined with the case conceptualization approach of TranS-C, script elicitation (i.e., reflection on and reorganization of the content and sequencing of habitual routines) [78] will be used to collaboratively derive the primary and secondary habit bundle(s) to be built and dismantled (for examples, see Table 2). Through this process, for each habit bundle, the participant’s current routine—including contextual cues, behaviors, and associated thoughts/feelings—will be elicited and recorded in detail. Then this information will be reviewed to identify unhelpful habitual behaviors to be dismantled. Each of the identified unhelpful behaviors will be assessed to (1) determine what contextual cues prompt it, (2) determine what is rewarding about it, and (3) select and describe a method (i.e., substitution, removing or curtailing the activity, or re-organizing the activity) to dismantle it. After this, an alternative habit bundle—consisting of a new sequence of behaviors—is developed. The alternative bundle incorporates recommendations from sleep and circadian science (i.e., adding helpful behaviors that support sleep and circadian functioning) and the previously determined method to dismantle the current unhelpful habitual behaviors (i.e., step 3 above; dismantling unhelpful habitual behaviors that may interfere with sleep and circadian functioning). To ensure that the alternative habit bundle(s) can be repeated daily, contextual cues will be identified to prompt the bundle(s) to be built, potential benefits/rewards will be identified, and anticipated barriers/obstacles will be assessed to support problem solving.

##### Motivational enhancement

Motivational enhancement will be incorporated session-by-session to identify motivational levers tied to personal interests, values, and motivations. These motivational levers or intrinsic rewards are key to elicit behavior change and build positive associations with the new habit bundle(s) [54, 67, 79]. Furthermore, motivational enhancement involves a review of the benefits and barriers of change [79] recognizing that many unhelpful sleep habits are often rewarding (e.g., texting with friends in bed) yet inconsistent with goals. Lastly, an implementation intention [80, 81] will be completed for each habit bundle to enhance the translation from goal intentions into action.

Sessions 4–9 will focus on refining the habit bundle(s) and supporting participants to achieve the sleep habit goals identified in the first three sessions. This will be achieved by assessing progress, continuing to incorporate the sleep and circadian science and habit science, addressing obstacles, reinforcing contextual cues, motivations and intrinsic rewards, and planning for maintenance and relapse education.

#### HABITs + Texts

In addition to the intervention outlined above (HABITs), the participants in HABITs + Texts will also receive the text message intervention for 6 weeks (after session 3 and through session 9; weeks 3–8 of the intervention). The text message intervention was derived using the science of habits [41, 47], learning theory [57], and the Behavior Change Wheel [67]. Each text message will be individualized to prompt engagement with the primary and secondary habit bundle(s) to be built and dismantled.

The text message intervention consists of three types of texts: cue texts, self-monitoring texts, and reward texts. The text messages will be sent using EZ Texting (© EZ Texting 2021), a secure platform for two-way text messaging that allows for automatic and scheduled messaging. The timing of the text messages is individualized and will incorporate the following principles: (1) the texts must not interfere with sleep (e.g., they will not be sent during the pre-sleep wind-down period), (2) the timing will be collaboratively derived (i.e., incorporating the timing of delivery that the participant feels would be most helpful), and (3) the timing will be as proximal as possible to the sleep promoting behavior that is targeted for habit formation, while respecting principles 1 and 2. The frequency or “dose” of the text messages follows a schedule informed by learning theory [57]. Details on the rationale and frequency of each type of text message (i.e., cue texts, self-monitoring texts, and reward texts) are outlined next and in Table 3.

**Table 3:** Frequency of texts per week

	Week 3^c^	Week 4	Week 5	Week 6	Week 7	Week 8
Cues^a^	7	4	4	3	2	2
Self-monitoring	7	7	7	7	7	7
Reward^b^	7	4	4	3	2	2

^a^ If the participant has both a primary and a secondary habit bundle, they will receive a separate cue for each habit bundle. ^b^ This is the text messaging reinforcement schedule when the reply to the self-monitoring text is “Y.” When the reply is “N,” we reply with a modified version of “That’s okay! Let’s try again tomorrow!”^c^ Week 3 starts after session 3 has been completed. In other words, week 8 ends once session 9 takes place

##### Cue texts

The cue texts are designed to establish and encode the contextual cue that will form the basis of the cue-behavior association that underpins the habit that participants will form [47, 48, 64]. For example, if 9:30 p.m. is the cue to the new habit (e.g., the wind-down routine), the text will say “It’s 9:30 p.m., I should start my wind-down routine and be in bed by 10:30 p.m.” Given that the salience of reminders decreases over time [82], the cue texts will include minor rewords across the intervention to maintain participants’ attention, while retaining the general structure and framework. For cue texts, the frequency will be as follows: after session 3 (week 3), every day; weeks 4 and 5, four randomly selected times per week; week 6, three randomly selected times per week; weeks 7 and 8, two randomly selected times per week.^3^ This “dose” of texting cues was selected to promote sufficient repetition for habits to form [66]. If the participant has both a primary and a secondary habit bundle, they will receive a separate cue for each habit bundle.

##### Self-monitoring texts

The self-monitoring texts are a form of progress monitoring and thus an intervention in and of itself [75, 76]. They are also used to determine when a reward text is warranted and reinforces habit formation. Specifically, participants are asked to reply with a one-letter text message, “Y” (yes) or “N” (no) if they completed the habit bundle(s) they were previously cued to complete (e.g., “Did you complete at least one of your goals last night? (Y/N) Did you complete at least one of your goals this morning? (Y/N)”). For self-monitoring texts, the frequency will be once daily for the entirety of the intervention.^4^ If the participant has both a primary and secondary habit bundle, one self-monitoring text which combines the two bundles will be sent.

##### Reward texts

The reward texts are designed to be reinforcing and enjoyable, with fresh content, including either a fun sleep fact/tip or an individualized motivational lever and end with a positive emoticon. In particular, to build positive associations with the new habit bundle(s) the motivational levers are highlighted to link the extrinsic reward (an engaging text message) to the participants’ intrinsic rewards (e.g., better sleep). This text (e.g., “Wonderful! Remember, by having a solid wind-down routine, I will have better sleep throughout the night.”) is sent when the reply to the self-monitoring text is “Y.” When the reply to the self-monitoring text is “N,” we reply with a modified version of “That’s okay! Let’s try again tomorrow!” For reward texts, when the reply to the self-monitoring text is “Y,” participants will initially (week 3) receive continuous reinforcement to rapidly establish a causal relationship between responses and outcomes. This will be followed by weeks 4–8 being divided between 50 and 33% reinforcement. This switch to partial reinforcement along with an “expanding-spaced” schedule was selected to promote resistance to extinction [58, 59]. The frequency of the rewards texts (only when the participant confirms engaging in the behavior) will be as follows: week 3, every day (to rapidly establish a causal relationship between responses and outcomes); weeks 4 and 5, up to four randomly selected times per week; week 6, up to three randomly selected times per week; weeks 7 and 8, up to two randomly selected times per week^3^. If the participant has both a primary and secondary habit bundle, one reward text which combines the two bundles will be sent.

### Measures

In addition to the measures below, a sociodemographics form is completed by participants at the pre-treatment assessment. Only measures that will be analyzed for the main aims of the study are reported below (see Aims section). The schedule for administering the measures is in Table 4. Unless otherwise specified, the measures below will be administered at the pre-treatment, post-treatment, 6-month follow-up, and 12-month follow-up assessments. To see which measure is associated with each outcome (i.e., utilization, habits, sleep and circadian functioning, and health-relevant domains) see Table 5.

**Table 4:** Timeline of measures

		**Pre-treatment**	**Treatment**	**Post-treatment**	**6-month follow-up**	**12-month follow-up**
	Sociodemographics	X				
**Primary**	Utilization Scale	X		X	X	X
	SRBAI-US	X		X	X	X
	CSHS	X		X	X	X
	CSM	X		X	X	X
	PROMIS-SRI	X		X	X	X
	PROMIS-SD	X		X	X	X
	Adapted WSAS Total Score	X		X	X	X
**Secondary**	Adapted SRHI – Primary Bundle		X	X	X	X
	PSQI	X		X	X	X
	Sleep Diary^a^	X	X	X	X	X
	Actigraphy^a^	X		X		
	DASS	X		X	X	X
	PROMIS-CF	X		X	X	X
	BSSS	X		X	X	X
	PROMIS-APS	X		X	X	X
	PHQ	X		X	X	X
	EMA Composite Risk Score^b^	X		X		
**Other**	Adapted SRHI – Secondary Bundle		X	X	X	X
	Adapted WSAS^c^	X		X	X	X
	Sleep Diary^d^	X	X	X	X	X
	Actigraphy^e^	X		X		
	Adapted Authenticity Scale	X		X	X	X
	Adapted CEQ^f^		X			
	Adverse Events Checklist			X		
	Proportion of Text Messages Read^g^		X			

*Note.* SRBAI-US Adapted Self-Report Behavioral Automaticity Index integrated with the Utilization Scale , CSHS Composite Sleep Health Score, CSM Composite Scale of Morningness, PROMIS-SRI PROMIS Sleep-Related Impairment Scale, PROMIS-SD PROMIS Sleep-Disturbance Scale, Adapted WSAS Adapted Version of the Work and Social Adjustment Scale, Adapted SRHI – Primary Bundle Adapted Self-Report Habit Index – Primary Bundle to Build, PSQI Pittsburgh Sleep Quality Index, DASS Depression, Anxiety, and Stress Scales, PROMIS-CF PROMIS-Cognitive Function Scale, BSSS Brief Sensation Seeking Scale, PROMIS-APS PROMIS-Ability to Participate in Social Roles & Activities Scale, PHQ Physical Health Questionnaire, EMA Ecological Momentary Assessment, Adapted SRHI – Secondary Bundle Adapted Self-Report Habit Index – Secondary Bundle to Build, Adapted CEQ Adapted Credibility Expectancy Questionnaire

^a^Night-to-night variability in the mid-point of sleep will be analyzed as a secondary outcome. ^b^ Includes five measures assessing the emotional, cognitive, behavioral, social, and physical health domains. ^c^ Individual items of this measure will be reported. ^d^ Remaining consensus sleep diary variables (i.e., total sleep time, bedtime, and wake-time). ^e^ Sleep parameters (i.e., sleep onset time, sleep offset time, and total sleep time). ^f^ Collected only at the second session of treatment. ^g^ Collected only at the last session of treatment

**Table 5:** Measures and corresponding outcome

		**Utilization**	**Habits**	**Sleep & Circadian**	**Health-Relevant Domains**				
					**Emotional**	**Cognitive**	**Behavioral**	**Social**	**Physical**
**Primary**	Utilization Scale	X							
	SRBAI-US	X	X						
	CSHS			X					
	CSM			X					
	PROMIS-SRI			X					
	PROMIS-SD			X					
	Adapted WSAS Total Score				X	X	X	X	X
**Secondary**	Adapted SRHI – Primary Bundle		X						
	PSQI			X					
	Sleep Diary^a^			X					
	Actigraphy^a^			X					
	DASS				X				
	PROMIS-CF					X			
	BSSS						X		
	PROMIS-APS							X	
	PHQ								X
	EMA Composite Risk Score^b^				X	X	X	X	X
**Other**	Adapted SRHI – Secondary Bundle		X						

*Note.* SRBAI-US Adapted Self-Report Behavioral Automaticity Index integrated with the Utilization Scale; CSHS Composite Sleep Health Score, CSM Composite Scale of Morningness, PROMIS-SRI PROMIS Sleep-Related Impairment Scale, PROMIS-SD PROMIS Sleep-Disturbance Scale, Adapted WSAS Adapted Version of the Work and Social Adjustment Scale, Adapted SRHI – Primary Bundle Adapted Self-Report Habit Index – Primary Bundle to Build, PSQI Pittsburgh Sleep Quality Index, DASS Depression, Anxiety, and Stress Scales, PROMIS-CF PROMIS-Cognitive Function Scale, BSSS Brief Sensation Seeking Scale, PROMIS-APS PROMIS-Ability to Participate in Social Roles & Activities Scale, PHQ Physical Health Questionnaire, EMA Ecological Momentary Assessment, Adapted SRHI – Secondary Bundle Adapted Self-Report Habit Index – Secondary Bundle to Build

^a^ Night-to-night variability in the mid-point of sleep will be analyzed as a secondary outcome. ^b^ Includes five measures assessing the emotional, cognitive, behavioral, social, and physical health domains

#### Primary measures

##### Utilization scale

The utilization scale is a self-report measure that will assess the extent to which participants have used the main sleep health behaviors that comprise HABITs over the past 7 days [39]. The 16 items will be rated using a 5-point Likert scale (0 = *never*, 4 = *always*). Two items were added to the original 14-item scale to assess dim lights during wind-down and bright lights in the morning. Examples of items rated include “I wind-down before bedtime” and “I stop using a screen-based device before bedtime.” A Utilization Score will be created by calculating the mean of all 16 items, where higher scores indicate greater utilization. In past studies, this measure had acceptable psychometric properties [39]. The psychometric properties of the Utilization scale will be reported in a supplement to the main report.

##### Adapted self-report behavioral automaticity index integrated with the utilization scale (SRBAI-US)

This measure was developed for this study to assess sleep health behavior habit formation. This measure was derived by combining the Self-Report Behavioral Automaticity Index [70] and the Utilization Scale [39], to measure the automaticity of each sleep health behavior item over the past 7 days. The 16 items on the utilization scale will each be rated using a 5-point Likert scale (0 = *never*, 4 = *always*) (e.g., “Deciding to wind-down before bedtime is something I do automatically”). An Automaticity Utilization Score will be created by calculating the mean of all 16 items, where higher scores indicate greater automaticity in utilization. The psychometric properties of the SRBAI-US will be reported in a supplement to the main report.

##### Composite sleep health score (CSHS)

The Composite Sleep Health Score will be used to capture overall sleep health for the complexity of sleep problems that are covered by HABITs [83]. It is defined as the sum of scores on six sleep health dimensions (each dimension dichotomized as 0 = *poor*, 1 = *good*): regularity (midpoint fluctuation), satisfaction (sleep quality question on PROMIS Sleep-Disturbance Scale), alertness (daytime sleepiness question on PROMIS Sleep-Related Impairment Scale), timing (mean midpoint), efficiency (sleep efficiency), and duration (total sleep time). All dimensions—except satisfaction and alertness—will be assessed via a sleep diary^5^ completed for 1 week, at each assessment timepoint. Information on the derivation of the Composite Sleep Health Score will be reported in a supplement to the main report. Total scores range from 0 to 6, where higher scores indicate better sleep health. Initial validity of this measure has been established [83].

##### Composite scale of morningness (CSM)

The CSM will assess the participants’ circadian rhythm preference (i.e., morningness or eveningness) via self-report [84]. The 13 items will each be rated using a combination of 4- and 5-point Likert scales. Total sum scores range from 13 to 55, where lower scores indicate greater eveningness. The coefficient alpha found in prior studies (0.87) indicates that the composite scale possesses excellent internal consistency reliability [84].

##### PROMIS Sleep-Related Impairment Scale (PROMIS-SRI)

The PROMIS-SRI will assess daytime impairment related to sleep problems over the past 7 days [85]. The eight items will each be rated on a 5-point Likert scale (1 = *not at all*, 5 = *very much*). T-scores (M = 50; SD = 10) will be calculated from the sum of the raw scores using scoring manuals obtained from healthmeasures.net, where higher scores indicate greater impairment (e.g., daytime sleepiness, difficulty concentrating). This measure has demonstrated excellent psychometric properties [85, 86].

##### PROMIS sleep-disturbance scale (PROMIS-SD)

The PROMIS-SD will assess disruption to sleep (e.g., restlessness, trouble staying asleep) over the past 7 days [85]. The eight items will each be rated on a 5-point Likert scale (1 = *not at all/never/very poor*, 5 = *very much/always/very good*). T-scores (M = 50; SD = 10) will be calculated from the sum of the raw scores using scoring manuals obtained from healthmeasures.net, where higher scores indicate greater disturbance. This measure has demonstrated acceptable reliability and validity [85, 86].

##### Adapted version of the work and social adjustment scale (Adapted WSAS)

The WSAS [87] was adapted for this study to assess the five health relevant domains: emotional, cognitive, behavioral, social, and physical domains. Example items include “Because of my sleep problems, I have difficulty managing my *moods and emotions* (for example: I feel more depressed, anxious, irritable, self-critical, worried, detached)” and “Because of my sleep problems, I have difficulty *getting along with friends or my romantic partner or the people I live with* (e.g., I get into conflict, I avoid going out, I don't feel connection with others, I feel alone, I am not satisfied with my social activities or my relationships).” The five items will each be rated on a 9-point Likert scale (0 = *not at all*, 8 = *very severely*). Total sum scores range from 0 to 40, where higher scores indicate worse outcome. The psychometric properties of the Adapted WSAS will be reported in a supplement to the main report.

#### Secondary measures

##### Adapted self-report habit index – primary bundle to build (Adapted SRHI—Primary)

The Adapted SRHI—Primary will assess the habit strength of the primary bundle to build at each session during treatment, post-treatment, 6-month, and 12-month follow-up assessments. The original Self-Report Behavioral Automaticity Index (SRBAI) [70] is a 4-item automaticity-specific adaptation of the full Self-Report Habit Index (SRHI) [88]. The four items of the original SRBAI plus two items from the full 12-item SRHI (“I do frequently” and “That belongs to my (daily, weekly, monthly) routine”) will be added to assess the frequency and relevance to self-identity, the two other primary proposed characteristics of habit, in addition to automaticity [70]. In total, six items will each be rated on a 5-point Likert scale (1 = *strongly disagree*, 5 = *strongly agree*). Total sum scores range from 6 to 30, where higher scores indicate greater habit strength.

##### Pittsburgh sleep quality index (PSQI)

The PSQI will assess sleep quality over the past month [89]. The 19 items will each be rated using a combination of 4-point Likert scale (0 = *not during the past month/very good/no problem at all*, 3 = *three or more times a week/very bad/a very big problem*) and integer responses. A global score will be derived by summing the seven component sub-scores. Global scores range from 0 to 21, where higher scores indicate worse sleep quality. This measure has demonstrated acceptable test–retest reliability and validity [89].

##### Sleep diary and actigraphy

The Sleep Diary and Actigraphy will be used to calculate night-to-night variability in the mid-point of sleep for the Composite Sleep Health Score. The Sleep Diary is a self-report adapted version of the consensus sleep diary [90], to track subjective sleep for 7 days. The sleep diary will be collected at all assessments and at each session during treatment. The Actigraphy (Actiwatch® GT9X Link; Philips Respironics) will assess movement samples in 60-s epochs over a 7-day period. Actigraphy will only be collected at the pre-treatment and post-treatment assessments. The intra-individual variability will be calculated using the estimated within-subject standard deviation [91].

##### Depression, anxiety, and stress scales (DASS)

The DASS will assess negative emotional states of depression, anxiety, and stress over the past 7 days [92], a component of the emotional health domain. The 21 items will each be rated on a 4-point Likert scale (0 = *did not apply to me at all*, 3 = *applied to me very much, or most of the time*). Three subscale scores for depression, anxiety, and stress will be summed (7 items for each subscale) and multiplied by 2, subscale scores range from 0 to 42, where higher scores indicate worse outcomes. Additionally, a total sum DASS score will be derived and range from 0 to 63. This measure has demonstrated acceptable internal consistency and concurrent validity [93]. This measure will also function as a convergent validity measure when assessing the psychometric properties of the Adapted WSAS.

##### PROMIS-cognitive function scale (PROMIS-CF)

The PROMIS-CF will assess participant-perceived cognitive deficits over the past 7 days [94], a component of the cognitive health domain. The six items will each be rated on a 5-point Likert scale (1 = *very often (several times a day)*, 5 = *never*). T-scores (M = 50; SD = 10) will be calculated from the sum of the raw scores using scoring manuals obtained from healthmeasures.net, where higher scores indicate higher cognitive functioning. This measure has demonstrated good psychometric properties [94]. This measure will also function as a convergent validity measure when assessing the psychometric properties of the Adapted WSAS.

##### Brief sensation seeking scale (BSSS)

The BSSS will assess sensation seeking, a dispositional risk factor for various problem behaviors [95], a component of the behavioral health domain. The eight items will each be rated on a 5-point Likert scale (1 = *strongly disagree*, 5 = *strongly agree*). Total sum scores range from 8 to 40, where higher scores indicate higher sensation seeking. This measure has demonstrated very good internal consistency [96]. This measure will also function as a convergent validity measure when assessing the psychometric properties of the Adapted WSAS.

##### PROMIS-Ability to participate in social roles & activities scale (PROMIS-APS)

The PROMIS-APS will assess participants-perceived ability to perform one’s usual social roles and activities [97], a component of the social health domain. The four items will each be rated on a 5-point Likert scale (1 = *always*, 5 = *never*). T-scores (M = 50; SD = 10) will be calculated from the sum of the raw scores using scoring manuals obtained from healthmeasures.net, where higher scores indicate higher ability for social functioning. This measure has demonstrated good evidence of criterion and construct validity [97]. This measure will also function as a convergent validity measure when assessing the psychometric properties of the Adapted WSAS.

##### Physical health questionnaire (PHQ)

The Physical Health Questionnaire will assess somatic symptoms over the past 4 weeks [98], a component of the physical health domain. The 15 items will each be rated on a 3-point Likert scale (0 = *not bothered at all*, 2 = *bothered a lot*). Total sum scores range from 0 to 30, where higher scores indicate worse symptoms. This measure has demonstrated good validity and reliability [99]. This measure will also function as a convergent validity measure when assessing the psychometric properties of the Adapted WSAS.

##### Ecological momentary assessment (EMA) composite risk score

EMA will index real-world functioning in the five health-relevant domains at the pre-treatment and post-treatment assessments. The measures will be collected over 7 days via text message two times a day on weekdays (morning and night) and four times a day on weekends (morning, night, and two random times between the morning and night) for a total of 18 instances across 7 days.^6^ The five measures below were adapted from prior research [34, 100] and will be used to derive composite risk scores of functioning in the five health domains: emotional, cognitive, behavioral, social, and physical. Composite risk scores will be calculated by standardizing the raw score (i.e., *z*-scoring) for each measure and then averaging the standardized scores from each respective health domain’s measure. In line with prior research, we will reverse-code summary scores for certain measures when appropriate such that all scores of the measures within a domain indicate the same direction (i.e., high scores indicate greater risk).

##### Emotional: adapted positive and negative affect schedule (Adapted PANAS)

The Adapted PANAS combines items from the I-PANAS-SF for adults [101] and a version derived from the short form of the Children’s PANAS [102] to assess positive affect (PA) and negative affect (NA) [103]. The 16 items will each be rated on a 5-point Likert scale (1 = *very slightly or not at all*, 5 = *extremely*). Scores will be separated into the PA and NA scores, where higher scores indicate more positive or negative affect, respectively. The Positivity Ratio will also be calculated by dividing the total positive emotions by the total negative emotions experienced [104].

### Cognitive: concentration, distractedness, and focus

To assess concentration, distractedness, and focus, three items will be rated on a 5-point Likert scale (1 = *very slightly or not at all*, 5 = *extremely*). Participants will be asked “At the moment you received the text message from us, what were you doing?” Participants will then be asked to rate their concentration, distractedness, and focus. The three items were adapted from previous research [105]. A total mean score will be calculated.

Behavioral: Eating, caffeine, alcohol, nicotine, marijuana, opioids, prescription and over the counter (OTC) stimulants, and sleep aids

To assess behaviors related to substances, including food/drinks [106] and drugs, participants will report their daily frequency and intake of each. For example, participants will be asked “At the moment you received the text message from us, were you drinking a beverage?” Participants will then be asked to indicate which drink(s) they were consuming. An average weekly frequency and intake of each substance will be calculated. Additionally, participants will be asked to list the use of additional psychoactive drugs (e.g., cocaine, heroin).

### Social: the positivity ratio

To assess social activity, three items will assess if the participant is with anyone at the time of the EMA. Additionally, the Positivity Ratio (see EMA for Emotion domain) will be calculated when the participant is alone, with a family member, or with a friend.

### Physical: activity and sedentary behaviors

To assess physical activity for the day, one item will be rated on a 2-point Likert scale (yes, no). The question, “Were you physically active today?” has been used in previous research [107, 108].

#### Other measures

##### Adapted self-report habit index – secondary bundle to build (Adapted SRHI—Secondary)

The Adapted SRHI—Secondary will assess the habit strength of the secondary bundle to build at each session during treatment, post-treatment, 6-month, and 12-month follow-up assessments. This measure is comprised of 1-item from the original Self-Report Behavioral Automaticity Index. Based on previous research [70], the 1-item selected, “I do automatically,” has been shown to have adequate face validity based on expert ratings. The 1-item will be rated on a 5-point Likert scale (1 = *strongly disagree*, 5 = *strongly agree*), where higher scores indicate greater habit strength.

##### Adapted version of the work and social adjustment scale items (Adapted WSAS items)

This measure is described above. It is also included as an “other outcome” as we will also report the five individual item scores.

##### Sleep diary and actigraphy

The Sleep Diary has been described above and will also be used to calculate additional consensus sleep diary variables, including total sleep time, bedtime, and wake-time, calculated separately for weekdays and weekends. Actigraphy (Actiwatch® GT9X Link; Philips Respironics) has been described above and will assess sleep parameters, including sleep onset time, sleep offset time, and total sleep time, calculated separately for weekdays and weekends.

##### Adapted authenticity scale

An adapted version from previous research [109] of the original Authenticity Scale [110] will assess state authenticity. The four items will each be rated on a 7-point Likert scale (1 = *strongly disagree*, 7 = *strongly agree*). Total mean scores range from 1 to 7, where higher scores indicate greater authenticity. This measure will function as a divergent validity measure when assessing the psychometric properties of the Adapted WSAS.

##### Adapted credibility expectancy questionnaire (Adapted CEQ)

The Adapted CEQ will assess the participants’ perception of treatment credibility and expected improvement at the second session during treatment. The 6-item Credibility Expectancy Questionnaire (CEQ) [111] was adapted to include the first four items, assessing the credibility factor fully and the first item of the expectancy factor. In total, four items will be rated via a combination of three 9-point Likert scales (1 = *not at all logical/not at all successful/not at all confident*, 9 = *very logical/very successful/very confident*) and one 0–100% scale. Scores from all items will be converted to standardized *z*-scores, and then a total sum CEQ score will be derived, in addition to separate sum scores for the credibility and expectancy factors. Higher scores indicate that the treatment was evaluated as more credible and expected improvement.

##### Adverse events checklist

The Adverse Events Checklist will assess adverse events experienced during the intervention at the post-treatment assessment. The 16 items, adapted for this study from prior research [112, 113], will each be rated on a 2-point Likert scale (yes, no).

##### Proportion of text messages read

At the final treatment session, participants were asked: “How often did you read the habit reminder text messages?” This item is rated on a 0–100% scale. This measure was added to the protocol 11 months into the study as a manipulation check.

### Procedure

We will recruit participants within the USA and Canada, with by far the most participants from the former. Recruitment efforts will include distribution of information about the study to doctors and health care clinics as well as advertisements in the community (e.g., outpatient medical and mental health clinics in the San Francisco Bay Area) and online (e.g., Facebook, Reddit, and Instagram). People who are interested in learning more will be directed to contact the UCB research staff directly by phone or email.

Prospective participants will verbally consent to an initial eligibility phone screen. If the participant is eligible to participate in the full study, they will be invited to the pre-treatment assessment. Informed consent will be obtained prior to starting the pre-treatment assessment via HIPAA-compliant DocuSign, mail, or in-person, if the participant prefers. The pre-treatment assessment will be completed virtually (i.e., phone or HIPAA-compliant Zoom). Informed consent and assessments will be conducted by assessors (i.e., UCB research staff). Assessors enter anonymized data collected from the pre-treatment assessment via a HIPAA-compliant Qualtrics survey.

Once the participant is enrolled, they will be randomly allocated to HABITs or HABITs + Texts (see Allocation section for more details). Both intervention arms include HABITs, which involve three 50-min individual weekly sessions followed by six 30-min individual weekly sessions. Sessions are completed virtually via HIPAA-compliant Zoom. Treatment is delivered by sleep coaches (i.e., UCB research staff who are trained and supervised by AGH, ERA, MD, and LDS). Only the HABITs + Texts group will receive the text messaging intervention. Text messages will be delivered for 6 weeks. The text messages are collaboratively written by the participant and the participant’s sleep coach (and reviewed and approved by AGH or MD). During treatment, sleep coaches will assist participants in completing treatment assessments using HIPAA-compliant Qualtrics.

At the conclusion of treatment, typically within 8–11 weeks, the post-treatment assessment will take place within 2 weeks of the final session. The follow-ups will take place 6 months and 12 months after the pre-treatment assessment. The post-treatment assessment, 6-, and 12-month follow-ups will be completed by assessors via phone or HIPAA-compliant Zoom, and anonymized data will be entered via HIPAA-compliant Qualtrics.

### Allocation

Participants will be randomized in a 1:1 parallel group design to each intervention arm through a computerized randomization sequence. Randomization is stratified by age (≤ 24, 25 +) and sex, as there is evidence that these variables can impact sleep and/or intervention outcomes [114, 115]. The planned stratified randomization is part of the generation of the randomization sequence. Randomization will be conducted by one project coordinator (i.e., UCB research staff). The assessors will be blind to the intervention arm of the participants.

### Sample size

The sample size was determined by power analysis (via G*Power 3.1). Two-tailed alpha of 0.05 with a Bonferroni-corrected alpha of 0.017 for each of the three aims. The effect size was drawn from prior research on text message health interventions (*d* = 0.60) [64, 116, 117]. To achieve 80% power, 128 participants will be needed to detect between medium-effect-size group differences. We added 20% to account for attrition, yielding 160 participants.

### Data management and dissemination

All participant-identifiable data will be saved by the assessment team on a secure password-protected and HIPAA-compliant website. All participants will be assigned identification numbers. These identification numbers will then be used to link anonymized data that is collected via password-protected and HIPAA-compliant Qualtrics. When collecting assessments, assessors will enter the data into HIPAA-compliant Qualtrics. Participant-identifiable data is not shared with outside entities during or after the trial.

A Data Safety Monitoring Board (DSMB) has been formed to help prevent and manage adverse events. The DSMB will be convened on a bi-annual basis for the first year and annually thereafter. For each report, the number of participants screened, the number of participants entered, the number of participants dropping out with the reasons for discontinuing, participant descriptive information, and the number of adverse events or serious events will be reported. All adverse events or serious events will be reported to CPHS (i.e., Institutional Review Board), the DSMB, and NICHD. However, if safety issues arise, this schedule will be changed to monthly meetings. For each report, minutes will be kept.

Organizations not directly involved in the trial (e.g., NICHD, DSMB, Institutional Review Board) have the right to audit and, if such a situation arises, will determine the frequency and procedures for auditing. The project management team will regularly audit the monthly enrollment as well as the completeness and quality of the data.

Results from the trial, as well as analysis code, will be shared via peer-reviewed publications, professional conference presentations, and a summary newsletter for participants. Other than the authors and compliance with data-sharing agreements stipulated by the National Institutes of Health, no other entities have contractual agreements to access the final dataset.

### Roles and responsibilities

This trial is supervised by the principal investigator (AGH), who manages the assessment team, the sleep coaches, and the data management team. The principal investigator will meet with members of each team regularly as needed in addition to daily email communication. The assessment team will be responsible for the informed consent process and conducting assessments. The sleep coaches will be responsible for delivering treatment, devising the text messages, and assisting participants in completing treatment assessments. The data management team will be responsible for downloading, collating, and analyzing the data. The trial sponsor is University of California, Berkeley.^7^ Other than ethical approval for the study, the sponsor has no role or ultimate authority in study design; collection, management, analysis, or interpretation of the data; writing of the report; or the decision to submit the report for publication. Finally, there are no other individuals or groups (e.g., Trial Steering Committee or Stakeholder and Public Involvement Group) overseeing the study.

### Changes to preregistration

This study was preregistered on clinicaltrials.gov (identifier: NCT05167695) on December 22, 2021. Since the study start date (May 2022), six updates have been made to clinicaltrials.gov: (1) EMA data was removed from the 6-month and 12-month follow-up due to participant burden and insufficient resources, (2) sessions 4 to 9 of treatment could not be completed in 20 min and thus were updated to 30-min sessions, (3) two additional outcome measures were added as it became clear that participants were typically working on at least two habit bundles (i.e., Adapted SRHI for the primary and secondary habit bundles), (4) an additional outcome was added as a manipulation check (i.e., Proportion of Text Messages Read), (5) the inclusion criteria for eveningness was clarified to distinguish “night-to-night variation in sleep and wake times” as opposed to “sleep/wake times”, and (6) we removed season as a moderator as there is little empirical evidence to support or negate the role of season in treatment response. This decision also served to help minimize multiple comparisons.

## Planned analyses

### Preliminary analyses and missing data

Data will be audited for quality and completeness. Missing or aberrant data will be verified for maximal data integrity. We will evaluate the distributions of the outcome variables and ensure that all the assumptions of planned analyses (e.g., linearity, normally distributed residuals) are met.

As per the power analysis described, we plan to recruit 160 participants, which allows for 20% attrition. Intent-to-treat analyses will use all available data via maximum likelihood estimation and produce valid inferences if attrition depends on intervention group or on previous outcomes for the same participant [118]. Additionally, sensitivity analyses may be conducted using multiple imputation to address missing data (e.g., dropouts, every item is not completed). If dropout is related to other variables, they will be included as predictors in the model to reduce any bias due to non-random missing data.

For Aims 1–3, all models will control for stratification factors (i.e., sex, age) used during the randomization procedure. Prior to the main analyses, appropriate statistical tests will be used to examine pre-treatment (i.e., baseline) differences between groups (e.g., education, current living arrangement, relationship status) to ensure comparability of the two randomized conditions (HABITs vs. HABITs + Texts) at pre-treatment and adequacy of the randomization. These tests will not be used to select covariates in the intent-to-treat analysis [119]. Instead, the potential influences of pre-treatment differences will be evaluated as moderators (described below in Exploratory Aim 1).

### Dropout

Per CONSORT figure recommendations [119], the *N* by stage of dropout will be reported for the following: dropout after randomization, dropout after treatment has been completed but prior to post-treatment, 6-month, or 12-month follow-up assessments.a

### Aims 1–3: Effectiveness outcomes of HABITs versus HABITs + Texts

For Aims 1–3, we will use intent-to-treat analyses, including all randomized participants as randomized in the analysis regardless of their adherence or completion of the assigned intervention. We will use multilevel modeling [120] to account for multiple observations over time (pre-treatment, post-treatment, 6-month, and 12-month follow-up) nested within participants. The level 1 equation captures within-person variation across time and will include dummy-coded time indicators (0 = pre-treatment, 1 = post-treatment, 2 = 6-month follow-up, 3 = 12-month follow-up) as predictors. The level 2 equation captures between-person variations in the intercept and slope of time and will include dummy-coded intervention condition (0 = HABITs, 1 = HABITs + Texts) and intervention-by-time interaction terms, as predictors. The parameters of interest will be significant intervention-by-time interactions at the 5% level (i.e., differences in intervention effects on change in outcomes from pre-treatment to post-treatment, pre-treatment to 6-month follow-up, and pre-treatment to 12-month follow-up). Significant intervention-by-time interactions indicate that the trajectory of change on outcomes is significantly different over time, comparing HABITs + Texts to HABITs. For Aim 1a, utilization of sleep health behaviors, as indexed by the Utilization Scale will be modeled as the continuous outcome variable. For Aim 1b, sleep health behavior habits, as indexed by the primary measure (SRBAI-US), secondary measure (Adapted SRHI – Primary), and other measure (Adapted SRHI – Secondary), will be modeled as the continuous outcome variables, separately. For Aim 2, sleep and circadian functioning, as indexed by primary measures (i.e., CSHS, CSM, PROMIS-SRI, and PROMIS-SD) and secondary measures (i.e., PSQI, Sleep Diary and Actigraphy [night-to-night variability in the mid-point of sleep]) will be modeled as the continuous outcome variables, separately. For Aim 3, the latent variables of health risk, as indexed by the primary measure (Adapted WSAS) and secondary measures (i.e., DASS, PROMIS-CF, BSSS, PROMIS-APS, PHQ, and EMA Composite Risk Score^8^), will be modeled as the continuous outcome variables, separately.

### Sensitivity analyses

Two sets of sensitivity analyses will be run to account for complexities that may be relevant to outcomes of Aims 1–3. One set of sensitivity analyses will be run to help account for the complexities related to the texting intervention. These analyses will be conducted to account for the proportion of text messages each participant read. We will also account for those who did not receive the messages due to a logistical or technical issue (e.g., technical issues with EZ Texting, mobile device issues). In the second set, sensitivity analyses will be conducted to account for (a) participants’ perceptions of the intervention credibility and expectancy and (b) adverse events experienced during the intervention. In other words, these analyses will test the effectiveness of HABITs in various participant experiences, as perception [111] and adverse experiences may impact intervention effectiveness [112, 113].

### Exploratory aim 1: mediators and moderators of intervention effects

Structural equation modeling [121] will be used to test whether sleep health behavior habit formation, as indexed by the SRBAI-US, mediate the effects of the intervention (HABITs vs. HABITs + Texts) on improving sleep and circadian functioning and health-relevant risk, as indexed by the primary measures for each. Bootstrapping procedure with 5000 replications will be used to test the statistical significance of the indirect effect from intervention condition to outcomes via the mediator [122]. The Benjamini–Hochberg procedure [123] will be used to correct for multiple testing on the outcomes (i.e., sleep and circadian functioning and health-relevant risk) [124]. Moderators will be assessed using a three-way interaction, intervention-by-time-by-moderator, in the multilevel model described in Aims 1–3. Each moderator and outcome will be tested in a separate model. Moderators will include age group (older adolescents 18–20 years of age vs. emerging adults 21–30 years of age), sex (sex assigned at birth; male, female, prefer not to say), and socioeconomic status (income; in 10,000 increment buckets [e.g., 0–10,000]).

### Exploratory aim 2: effectiveness outcomes of HABITs across HABITs and HABITs + Texts

Should the HABITs versus HABITs + Texts intervention effects (Aims 1–3) or the within-group pre-post-treatment effects of HABITs be non-significant we will conduct exploratory analyses combining the two randomized groups (HABITs and HABITs + Texts) to evaluate if HABITs (regardless of whether the text message intervention is added) is associated with an improvement in the utilization of sleep health behavior, sleep health behavior habits, sleep and circadian outcomes, and the five health-relevant domain outcomes in the short (post-treatment) and longer term (6-month and 12-month follow-up), relative to pre-treatment. Multilevel modeling [120] will be used to account for multiple observations over time (pre-treatment, post-treatment, 6-month, and 12-month follow-up) nested within participants. The fixed component of the model captures within-person variation across time and will include dummy-coded time indicators (0 = pre-treatment, 1 = post-treatment, 2 = 6-month follow-up, 3 = 12-month follow-up). The model will control for stratification factors (i.e., sex, age) used during the randomization procedure. The parameter of interest will be significant coefficient of time at the 5% level. Significant coefficient of time indicates that the combined intervention effect is significantly different across time, with pre-treatment as the reference group. Specifically, we will test change in outcomes from pre-treatment to post-treatment, pre-treatment to 6-month follow-up, and pre-treatment to 12-month follow-up. Each outcome, utilization of sleep health behaviors, sleep health behavior habits, sleep and circadian functioning, and the latent variable of health risk, as indexed by the primary measures, will be modeled as the continuous outcome variables.

## Discussion

This study aims to evaluate the Habit-based Sleep Health Intervention (HABITs) and whether adding a text message intervention helps young adults to form habits that modify the psychosocial and behavioral contributors to eveningness. By integrating the science on habits and learning theory with behavior change interventions, the aim is to help recipients turn new behaviors into habits and thereby increase the benefits of the intervention in both the short and longer term [42]. Additionally, the text message intervention has great potential because it is a low-cost, efficient, and potent intervention that may help habit formation and bolster the maintenance of behavior change [62–64]. We will investigate if this approach improves the utilization of sleep health behaviors, sleep health behavior habits, sleep and circadian functioning, and functioning in five health-relevant domains.

The findings have the potential to advance knowledge and address several research areas and priorities. First, this study will demonstrate whether leveraging the science of habits can be translated into effects on sleep and circadian functioning and health-relevant outcomes. Importantly, the habit formation approach is potentially “transdiagnostic” (relevant to a broad range of problems) and “pantreatment” (relevant to a broad range of types of treatment) [42]. Thus, evaluating this strategy may help other scientists improve interventions and outcomes for a variety of populations and settings. Second, this study will contribute to literature evaluating the use of a low-cost and efficient text message intervention, grounded in learning theory, to bolster habit formation and maintenance. Third, by focusing on participants with an eveningness chronotype, the present study will add to the literature on how addressing eveningness may improve outcomes in the short and longer term. The latter is particularly important given eveningness’ adverse health consequences and the limited research on interventions to address it. Fourth, this study will contribute to knowledge on the processes of behavior change during emerging adulthood, which is often marked by a shift in priorities toward self-sufficiency and personal responsibility. Therefore, advancing the understanding of processes related to the formation and maintenance of helpful behaviors is particularly important for the developmental period spanned by the study sample.

These potential contributions should be considered alongside the protocol’s methodological limitations. First, for assessing the five health-relevant domains, an extensive search of health measures (e.g., SF-36, PhenX, WHODAS, PROMIS, neuro-QL, BRIEF, GHQ) resulted in no single measure that covered all five health domains in a developmentally appropriate way. Hence, the Work and Social Adjustment Scale [87] was adapted to serve as a single measure assessing the five health domains (Adapted WSAS). In addition to the Adapted WSAS, each domain will be assessed with a different measure and through the EMA Composite Risk Score. These measures will also function as a measure of convergent validity when assessing the psychometric properties of the Adapted WSAS, which will be reported in a supplement to the main report. Second, as there is evidence that the large age range of participants (18–30 years of age) and sex can impact sleep and/or intervention outcomes [114, 115], the content and implementation method for HABITs will be adapted [125–127] by the sleep coach, with careful training and supervision, using an individualized case formulation [76] to determine the amount of time spent on each component and the style of delivering each component. Third, adding a placebo text arm to control for receiving a text was considered. However, the pilot study and focus groups indicated that placebo texts detracted from habit formation and were “annoying” to the participants. Additionally, including a placebo text arm would require a larger sample and more resources.

In sum, this study has the potential to advance knowledge on: (a) the value of leveraging the science of habits and learning theory in behavior change interventions, (b) the use of a low-cost and efficient text-based intervention for habit formation and maintenance, (c) interventions addressing eveningness and the impact on a range of outcomes, and (d) processes related to behavior change during an important and understudied stage of development (i.e., emerging adulthood).

## Trial status

Protocol version 1, December 9, 2021. Data collection started in May 2022 and will continue through July 2026. Recruitment started in May 2022 and is projected to finish in December 2025.

## Supplementary Information

Supplementary Material 1

## Data Availability

Other than the authors and compliance with data-sharing agreements stipulated by the National Institutes of Health, no other entities have contractual agreements to access the final dataset.

## Abbreviations

TranS-C: Transdiagnostic Intervention for Sleep and Circadian Dysfunction
HABITs: Habit-based Sleep Health Intervention
HABITs + Texts: HABITs plus Text Message Intervention
SRBAI-US: Adapted Self-Report Behavioral Automaticity Index integrated with the Utilization Scale
CSHS: Composite Sleep Health Score
CSM: Composite Scale of Morningness
PROMIS-SRI: PROMIS Sleep-Related Impairment Scale
PROMIS-SD: PROMIS Sleep-Disturbance Scale
Adapted WSAS: Adapted Version of the Work and Social Adjustment Scale
Adapted SRHI – Primary: Adapted Self-Report Habit Index – Primary Bundle to Build
SRBAI: Self-Report Behavioral Automaticity Index
SRHI: Self-Report Habit Index
PSQI: Pittsburgh Sleep Quality Index
DASS: Depression, Anxiety, and Stress Scales
PROMIS-CF: PROMIS-Cognitive Function Scale
BSSS: Brief Sensation Seeking Scale
PROMIS-APS: PROMIS-Ability to Participate in Social Roles & Activities Scale
PHQ: Physical Health Questionnaire
EMA: Ecological Momentary Assessment
OTC: Over the counter
Adapted PANAS: Adapted Positive and Negative Affect Schedule
PA: Positive affect
NA: Negative affect
Adapted SRHI – Secondary: Adapted Self-Report Habit Index – Secondary Bundle to Build
Adapted WSAS items: Adapted Version of the Work and Social Adjustment Scale items
Adapted CEQ: Adapted Credibility Expectancy Questionnaire
UCB: UC Berkeley
DSMB: Data Safety Monitoring Board
NICHD: Eunice Kennedy Shriver National Institute of Child Health and Human Development

## Footnotes

1 Depending on cancellations and rescheduled sessions, the intervention can be completed in up to 11 weeks.

2 Since the intervention can be completed in up to 11 weeks, the text intervention can range from 6 to 8 weeks.

3 If the intervention surpasses 8 weeks due to scheduling or cancellations, the texts will continue to be administered at two randomly selected times per week until the ninth session is completed.

4 If participants do not respond to the self-monitoring texts, one reminder per day will be sent to elicit a response and, if applicable, address any logistical or technical problems. If there are no responses after three consecutive days of reminders, support with troubleshooting will occur in the next session.

5 In rare cases, during an assessment timepoint, questions about sleep–wake patterns over the past 7 days (e.g., “Over the past week, what time have you usually woken up in the morning?”) may be used when a sleep diary is not available.

6 If the participant does not respond, we send reminders and troubleshoot technology errors. If participants have a low completion rate, we may ask them to complete EMA for another week.

7 Contact information for Sponsor: + 1(510) 642–6000; University Avenue and Oxford Street, Berkeley, CA 94702.

8 Includes five measures assessing the emotional, cognitive, behavioral, social, and physical health domains.
